# Failure modes and mitigations for Bayesian optimization of neuromodulation parameters 

**DOI:** 10.1088/1741-2552/ade189

**Published:** 2025-06-13

**Authors:** Evan M Dastin-van Rijn, Alik S Widge

**Affiliations:** Department of Psychiatry and Behavioral Sciences, University of Minnesota, Minnesota, MN, United States of America

**Keywords:** neuromodulation, bayesian optimization, simulation

## Abstract

*Objective.* Precision medicine holds substantial promise for tailoring neuromodulation techniques to the symptomatology of individual patients. Precise selection of stimulation parameters for individual patients requires the development of robust optimization techniques. However, standard optimization approaches, like Bayesian optimization, have historically been assessed and developed for applications with far less noise than is typical in neuro-psychiatric outcome measures and with minimal focus on parameter safety. *Approach.* We conducted a literature review of individual effects in neurological and psychiatric applications to build a series of simulated patient responses of varying signal to noise ratio. Using these simulations, we assessed whether existing standards in Bayesian optimization are sufficient for robustly optimizing such effects. *Main results.* For effect sizes below a Cohen’s d of 0.3, standard Bayesian optimization methods failed to consistently identify optimal parameters. This failure primarily results from over-sampling of the boundaries of the space as the number of samples increases, because the variance on the edges becomes disproportionately greater than in the remainder of parameter space. Using a combination of an input warp and a boundary avoiding Iterated Brownian-bridge kernel we demonstrated robust Bayesian optimization performance for problems with a Cohen’s d effect size as low as 0.1. *Significance.* Our results demonstrate that caution should be taken when applying standard Bayesian optimization in neuromodulation applications with potentially low effect sizes, as standard algorithms are at high risk of converging to local rather than global optima. Mitigating techniques, like boundary avoidance, are effective and should be used to improve robustness.

## 1. Introduction

The human brain is a complex, interconnected system with considerable variability between individuals. This is especially true for diseases of the brain; neurological and psychiatric diseases have substantial variation in symptoms, timecourse, and treatment efficacy that arises from a complex interplay of risk genes, dynamic biological determinants, and environmental factors [1]. Addressing this individual variability is the focus of precision medicine and will be integral to improving current ineffective treatments and reducing disease burden [2, 3]. Neuromodulation is an especially promising technique for applying precision medicine due to the wide array of tunable parameters including current dose, frequency, pattern, and location. However, for precision medicine to be effective in neuromodulation applications, it will be necessary to develop methods to rapidly identify the treatment parameters that would be most effective for an individual patient [4].

The primary difficulty with developing precision neuromodulation is that the connection between an individual’s unique background and their specific disease symptomatology is often a black box problem [5]. Extensive screens of physiological, behavioral, and symptom measures have enabled ‘big data’ and artificial intelligence methods to make extensive strides towards relating individual patient data to specific treatments [6, 7]. However, these approaches focus on inferring the treatment for a new patient based on experience with prior patients. While larger, more multimodal datasets will increasingly help to address patient heterogeneity [8], the exact parameters or dosage for an individual patient will inevitably differ somewhat from any predictive model [9]. Truly optimizing a treatment for an individual patient requires an algorithm that can test treatment parameters, obtain a response, and select future parameters based on a balance of exploration and exploitation specific to the patient.

**Figure 1.**
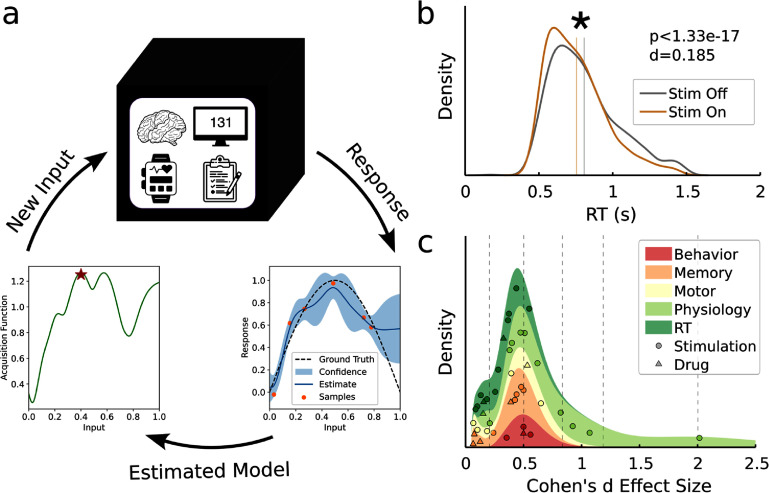
Feasibility of continuous optimization for therapeutic applications in neuroscience. (a) We frame this process as a black box problem where the patient continuously generates trials of data from any of a variety of sources including physiological measurements, psychophysical task outcomes, or surveyed metrics. These responses are fed to a Gaussian process regression which estimates a model of the patient’s response across the possible inputs. The next input to test is generated by optimizing a balance of exploitation and exploration via a heuristic (acquisition function). This process continuously repeats until an optimal input is identified. (b) Distributions of reaction time during a cognitive control task Widge *et al* [23] for active (stim on) and inactive (stim off) stimulation. This effect was significant (*p* < 1.33 × 10^−17^) but had a small effect size (*d* = 0.185). (c) The effect size in (b) is not abnormal with most experiments reporting within subject effects having comparable effect sizes. Generally, physiological measurements showed the largest effect sizes but the majority of studies reported effect sizes below the canonical ‘medium’ effect (*d* = 0.5). These are smaller effects than typically addressed by optimization literature, raising new challenges.

In essence, this is a global function optimization problem where the minimum or maximum of a function (the patient response) is queried based on selected locations in the parameter space (treatment parameters). In the case of neuromodulation, the function being optimized could be a physiological measurement, performance on a behavioral task, or symptom self-report. The parameters, correspondingly, might be intensity, temporal pattern, or location of stimulation. Bayesian optimization is a particularly effective approach for global optimization that has already shown promise in neurological and psychiatric applications [10–16]. Bayesian optimization relies on a surrogate model of the response surface (typically a Gaussian process) (GP) which is constructed from available observations of the function [17, 18]. The parameters at which the function is next evaluated are chosen as the location that maximizes an acquisition function-a heuristic balancing the predicted value of the function at each location with the uncertainty in the prediction [19–22]. This three step process of evaluation, model fitting, and parameter selection would repeat until the optimal treatment parameters are identified with sufficient certainty (figure 1(a)).

However, the existing literature applying and assessing Bayesian optimization is predominantly focused on applications with relatively high signal to noise ratio [24–26]. While this is an accurate assumption in fields like materials science, robotics, and machine learning, most measurements from the brain and related neural systems are defined by substantial noise [27]. As a result, the within-subject effects that would be leveraged by Bayesian optimization are characterized by small changes relative to the magnitude of the noise. While such effects may seem modest, changes of 5%–10% can be significant and behaviorally meaningful. As an example of such an effect, consider the DBS study conducted by Widge *et al* (figure 1(b)) [23]. In this study, reaction time was used as a readout of cognitive control and was shown to decrease with stimulation. Bayesian optimization could feasibly be used to identify the DBS parameters that would optimize this outcome [12]. However, the effect, while highly significant (*p* < 1.33 × 10^−17^), is visibly small relative to the noise resulting in a small Cohen’s d effect size of 0.185. This effect size issubstantially smaller than what is typically addressed by Bayesian optimization and it has not been conclusively demonstrated that the technique is effective at these noise levels.

Additionally, much of the existing Bayesian optimization literature focuses on optimization independent of potential safety concerns associated with the choice of parameters [28, 29]. In patient facing applications like neuromodulation, safety cannot be ignored during the optimization process. Slightly too much current in the wrong location is sufficient to initiate seizures or off-target motor and cognitive effects [30–32]. Sensitivity to treatment parameters is also patient-specific, necessitating an optimization approach that is compatible with complex safety boundaries rather than the simple hypercubes that are typically employed in Bayesian optimization.

To investigate the potential of using Bayesian optimization for neuromodulation in neurological and psychiatric applications, we conducted a set of simulations over a variety of potential response surfaces, problem effect sizes, and safety boundaries informed by a meta-analysis of typical within-subject effects of neuro-stimulation and pharmaceuticals. We found that standard methods for Bayesian optimization perform poorly for problems with low effect sizes, even though low effect size problems are the majority of the applications in neurology and psychiatry. We identified that this poor performance resulted from excessive variance on the boundaries of parameter space and a corresponding over-sampling of these boundaries. Methods that addressed this variance and boundary over-sampling substantially improved optimization performance suggesting that Bayesian optimization, with appropriate adjustments, can be an effective technique for optimizing treatments for nervous system disorders.

## 2. Methods

### 2.1. Effect size meta-analysis

To ensure our simulations accurately represented typical problems in the field, we conducted a meta analysis of the within-subject effects of neuro-stimulation and pharmaceuticals on a variety of clinically relevant outcomes.

Our review focused on two primary questions:

1) What are the typical within-subject effect sizes of stimulation and drug interventions for psychiatric and neurological applications?
2) Do these effect sizes depend on the outcome type?

We searched using Google Scholar for articles reporting within-subject measures of mean change and standard deviation (or directly reporting within-subject effect size) for a neural-related outcome. Effect size was computed using Cohen’s d [33]. We considered articles published in any indexed year. We did not specify which outcome measures to consider in order to broadly assess reported effects on a variety of relevant brain and behavioral metrics. We only included effects that were reported as statistically significant (*p* < 0.05). For articles that reported multiple measurements for the same outcome, we only included the one with the largest effect size [34–46]. Articles with more than one outcome type or intervention were treated as separate studies [35, 37, 39, 41, 44, 47–50]. After reviewing the articles, we divided the outcomes into five categories to identify any potential differences: general behavior (accuracy, performance, etc.) [40, 43, 51, 52], memory [35, 38, 41, 42, 47, 53], motor [44, 49, 50, 54], physiology (brain activity, pupillometry, skin conductance response, etc.) [35, 37, 39, 44–46, 48, 55, 56], and reaction time [23, 34–37, 49, 50, 57–61].

### 2.2. Simulation approach

**Figure 2.**
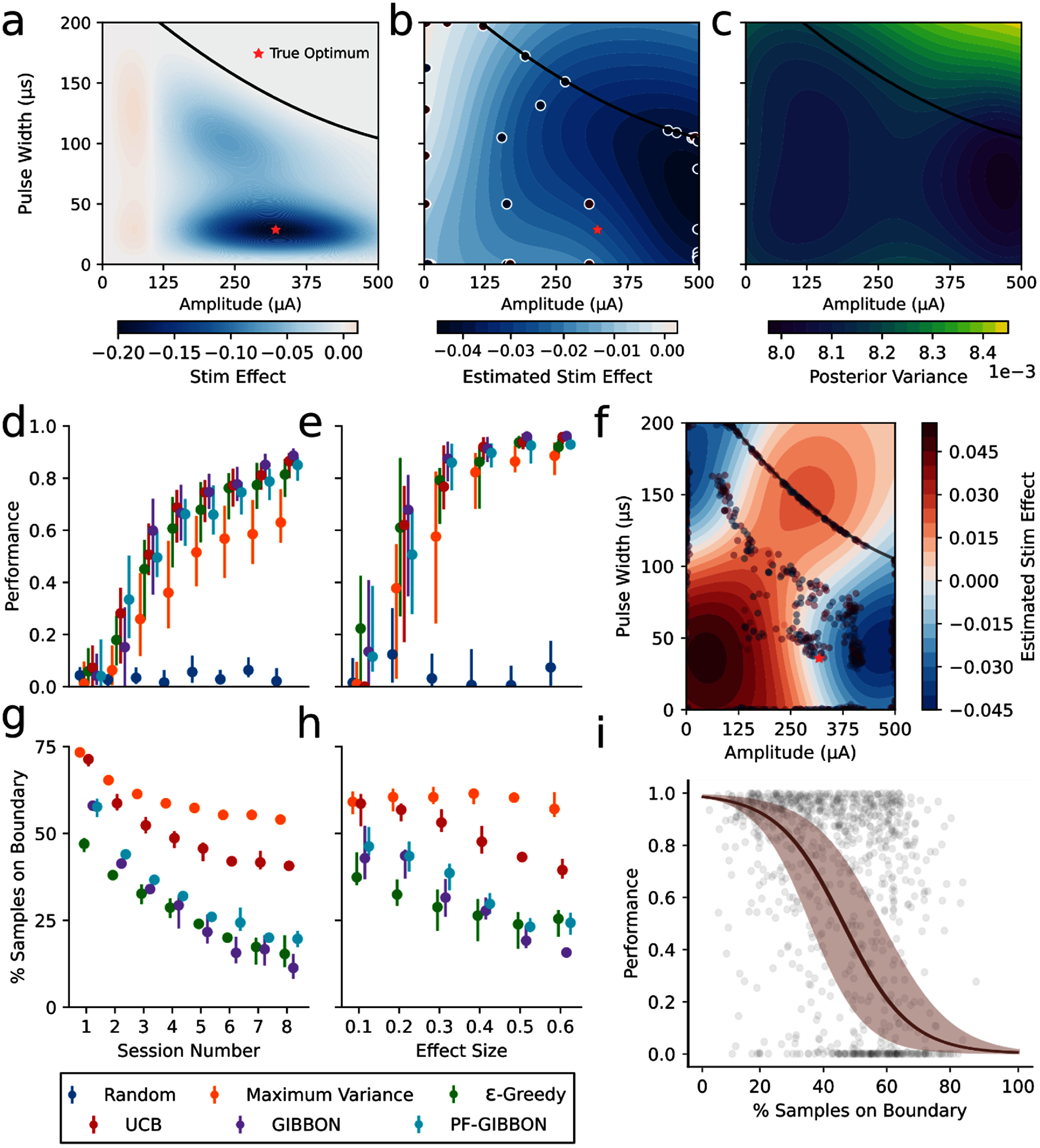
Assessing Bayesian optimization performance for a typical simulated problem. (a) Example simulated ground truth surface used to generate samples as a function of input parameters. (b), (c) Gaussian process posterior mean (b) and variance (c) after 30 example samples for the problem shown in (a). Variance is high in regions with few samples (for example, beyond the safety boundary) and low where there are many samples. (d), (e) Performance of Bayesian optimization for five different acquisition functions, compared against random selection. Performance was calculated as the ratio between the effect of the learned optimal parameters to the ground truth optimal parameters. (d) Performance increased with the number of completed sessions, with all acquisition functions outdoing random selection after a single session. (e) Performance was substantially reduced for low effect size problems with UCB and variance maximization failing to outdo random sampling for the lowest effect size. (f) Example completed optimization for the GIBBON acquisition function showing excessive sampling on the boundaries of the parameter space. (g), (h) Percentage of samples on the boundary of the space for the four acquisition functions. (g) The number of samples on the boundary decreased with each session for all acquisition functions. (h) The boundary was most oversampled for problems with a low effect size. (i) Logit transformed linear regression between the percentage of samples on the boundary and final optimizer performance across the five acquisition functions. 95% confidence interval on the fit is indicated by the shaded region. Problems with a high degree of boundary sampling were significantly more likely (*p* < 7.08 × 10^−50^) to have poor optimization performance.

To assess the impact of effect size on the efficacy of Bayesian optimization, we generated simulated response surfaces for a two-parameter optimization problem based on our prior work using DBS to modulate cognitive control [23, 34, 57] (example surfaces are shown in figure 2(a) and supplemental figure 1). Our two parameters represent the amplitude and pulse width of the stimulation and respectively ranged from 0 to 500 *μ*A and 0–200 *μ*s corresponding to a reasonable range from prior experiments in rodents [57]. The range of values is arbitrary for the optimization methods but is useful for assessing if the curvature and shape of the surface would be reasonable in practice. To produce the surfaces, 10 sinusoids were generated for each parameter ($x$ or $y$) with amplitudes ($A$) ranging from −0.5 to 0.5 and frequencies ($F$) ranging from 0 to 1 cycle over the length of the parameter space. All sinusoids for both parameters were summed together to produce the full 2D surface maps (${f^o}$). The full equation for generating the surfaces is described below:

\begin{align*}{f^o}\left( {x,y} \right)&amp; = \mathop \sum \limits_{i = 1}^{10} {A_{i,0,0}}{\text{sin}}\left( {2\pi {F_{i,0}}x} \right) + {A_{i,0,1}}{\text{cos}}\left( {2\pi {F_{i,0}}x} \right)\nonumber\\ &amp; \quad + \mathop \sum \limits_{i = 1}^{10} {A_{i,1,0}}{\text{sin}}\left( {2\pi {F_{i,1}}y} \right) \nonumber\\ &amp; \quad+ {A_{i,1,1}}{\text{cos}}\left( {2\pi {F_{i,1}}y} \right)\end{align*}

\begin{equation*}A\sim {\text{Uniform}}\left( { - {\text{0}}{\text{.5,0}}{\text{.5}}} \right)\end{equation*}

\begin{equation*}F\sim {\text{Uniform}}\left( {0,1} \right)\end{equation*}

A quadratic safety boundary was also generated for each simulation according to the following equation:

\begin{equation*}y = \frac{{{{\left(x - h\right)}^2}}}{a} + k.\end{equation*}

A quadratic boundary was chosen for this particular simulation as an approximation of a charge delivery curve. The tolerability of stimulation parameters is typically a function of the charge delivered which would be proportional to the product of amplitude and pulse width (an inverse relationship) [62]. We found that a simulation using an inverse equation as a boundary required numerical rather than analytic solutions which would have substantially slowed the analysis. To address this bottleneck, we used a quadratic approximation which we found mimicked an inverse boundary over a reasonable range of values (supplemental figure 2) while allowing for an analytic solution.

The parameters for the boundary were randomly sampled according to the following equations:

\begin{equation*}h\sim {\text{Uniform}}\left( {500,1500} \right)\end{equation*}

\begin{equation*}k\sim {\text{Uniform}}\left( {0,200} \right)\end{equation*}

\begin{equation*}a\sim {\text{Uniform}}\left( {\frac{{{{\left(500 - h\right)}^2}}}{{200 - k}},\frac{{{h^2}}}{{200 - k}}} \right).\end{equation*}

The portions of the simulated surface near the left, bottom, and safety boundaries were sigmoidally scaled according to the distance ($d$) from the corresponding boundary to mimic negligible effects for zero output and expected decreases in response efficacy along the safety bound. The exact equation for this scaling function ($s$) is shown below:

\begin{equation*}s\left( d \right) = \left\{ {\begin{array}{*{20}{c}} 0&amp;{d &lt; 0} \\ {\frac{{{d^{1.5}}}}{{{{\left( {0.2 - d} \right)}^{1.5}}}}}&amp;{0 &lt; d &lt; 0.2} \\ 1&amp;{d &gt; 0.2} \end{array}} \right.\end{equation*}

This produces a new, modified response surface (${f^*}$) according to the following equation, where $D$ is the set of boundary distances:

\begin{equation*}{f^*}\left( {x,y} \right) = {f^o}\left( {x,y} \right)\mathop \prod \limits_d^D s\left( d \right).\end{equation*}

The value and location of the maximum and minimum of each surface was identified using the *scipy* function *fmin_l_bfgs_b* [63]. Surfaces were generated to always have the absolute value of the minimum exceed the maximum.

Using the values for the maximum and minimum, each surface was rescaled to have a minimum value corresponding to the effect size (${\text{ES}}$) for the problem. This surface (${f^R}$) is described by the following equation:

\begin{equation*}{f^R}\left( {x,y} \right) = \frac{{{f^*}\left( {x,y} \right)}}{{\left| {{\text{min}}\left( {{f^*}\left( {x,y} \right)} \right)} \right|}}{\text{ES}}{\text{}}.\end{equation*}

We modeled effect sizes of 0.1–0.6 in steps of 0.1 by appropriately rescaling the response surfaces and setting the sampling noise to have a standard deviation of 1. Therefore, an individual sample ($f$) from the response surface would be generated according to the following equation:

\begin{equation*}f\left( {x,y} \right) = N\left( {{f^R}\left( {x,y} \right),1} \right)\end{equation*}

20 simulated surfaces were generated per effect size resulting in a total of 240 simulated optimization problems. Examples of surfaces with different optimal locations, presence of multiple local minima, and various safety bounds are shown in figure 2(a) and supplementary figure 1.

### 2.3. Standard Bayesian optimization

Bayesian optimization is a popular method for global optimization that is particularly effective for problems where each evaluation of the function is expensive to compute [17]. This global optimization problem can generally be expressed as follows:

\begin{equation*}\frac{{{\text{max}}}}{{x\in A}}f\left( x \right)\end{equation*}

where $A \subset {\Re ^d}$ is the feasible space. The overall approach of Bayesian optimization is to sequentially sample the parameter space at likely or informative locations of the global optimum by taking into account not only the predictions of some surrogate model but also the uncertainty in the predictions. We began each of our optimization protocols by sampling 6 points in the left corner of the parameter space ([0,0, 150, 0, 300, 0, 300, 50, 1, 50, 0]). We then fit a GP as our choice of surrogate model to these initial points. At each iteration, this GP was updated based on the acquired data and an acquisition function was computed from the mean and variance of the GP and maximized to choose the next sampled point according to a heuristic balance of exploration and exploitation. Every 150 trials, the hyperparameters of the GP were re-optimized as this is a more time consuming problem that might be done intermittently after a patient has finished a treatment session, behavioral task, or sequence of measurements. A total of 8 blocks of 150 trials were run for each optimization protocol.

For our GP, we chose a Matern 5/2 kernel with zero mean and fixed output noise of 1 standard deviation for a standard multivariate Gaussian likelihood using the *gpytorch* package [64]. Remaining hyperparameters (lengthscale and output scale) were optimized using the *fit_gpytorch_mll* function which is part of the *botorch* package [65]. Acquisition functions were optimized using the *optimize_acqf* in *botorch* with a nonlinear constraint corresponding to the true safety bound from the simulated response surface. For some applications, this bound would be known in advance (e.g. charge density limits) however many robust methods have been described in the literature to identify the safe regions of parameter space in cases where bounds are unknown [28, 29, 66]. We ran our optimization simulations using five different acquisition functions: maximum variance, upper confidence bound (UCB), general-purpose information-based Bayesian optimization (GIBBON), *ϵ*-greedy, and pareto front-GIBBON. These acquisition functions were chosen based on their robustness to noise and prior successes in similar applications [12]. Maximum variance is a purely exploratory acquisition function where at each iteration the point in parameter space with the greatest variance is selected. This acquisition function serves as a useful benchmark for determining if the actual optimization process yields greater performance than naively exploring unseen parameters [20]. UCB is an acquisition function that explicitly balances exploration and exploitation by computing a weighted sum of the GP posterior mean and standard deviation:

\begin{equation*}UCB\left( {x,y} \right) = \mu \left( {x,y} \right) + \sqrt {{\beta _t}} \sigma \left( {x,y} \right)\end{equation*}

where ${\beta _t}$ is scaled as the optimization progresses to ensure a theoretical zero-regret bound [19]:

\begin{align*}\beta_\text{t} &amp; = 2\text{log}\left({\text{t}^3}\frac{\pi^2}{3\text{p}}\right)\nonumber\\ p &amp; = 0.01.\end{align*}

GIBBON is an extension of the max-value entropy search acquisition function which identifies points in the parameter space that would maximally reduce the uncertainty regarding the optimal value of the outcome [21, 22]. GIBBON employs an approximation scheme that allows it to be more computationally-lightweight than standard max-value entropy search while having comparable performance and noise tolerance. *ϵ*-greedy is an acquisition function that randomly selects points in parameter space that have a pareto optimal tradeoff between exploration (posterior variance) and exploitation (posterior mean) whereas standard acquisition functions like UCB and expected improvement (EI) determine a fixed heuristic tradeoff [20]. At each iteration, *ϵ*-greedy will either select the predicted, optimal location in parameter space with probability $p$ or a random point on the exploration-exploitation pareto front with probability $1 - p$. For the purposes of our simulations, we fixed $p$ to a constant value of 0.1. At each iteration, points on the exploration-exploitation pareto front were identified using the NSGA-II algorithm implemented in the *pymoo* package [67, 68]. Based on the observation that GIBBON would occasionally converge to a solution too quickly without adequately exploring the parameter space, we implemented a combination of *ϵ*-greedy and GIBBON that would use the parameters suggested by GIBBON with probability $p$ or a random point on the exploration-exploitation pareto front with probability $1 - p$. For the purposes of our simulations, we fixed $p$ to a constant value of 0.3.

The effectiveness of each optimization was evaluated by computing a performance metric at the end of each session. Performance was determined by evaluating the ground truth response surface at the optimal parameters according to the GP posterior mean and normalizing by the effect size for the problem. The optimal parameters from the GP were identified by maximizing the *posterior mean* acquisition function in *Botorch*. This metric is analogous to a normalized version of inverse regret which is commonly employed to evaluate the efficacy of acquisition functions in Bayesian optimization. We also evaluated boundary over-sampling by computing the percentage of samples that were on the boundary of the space or the safety boundary. High values for this percentage would indicate an optimization run where an excessive number of samples were in uninformative areas of parameter space. Performance and boundary over exploration were evaluated across sessions as a function of the session number or effect size. Confidence intervals on median performance were computed using bootstrapping with 1000 resamples via the *bootstrap* method in *scipy* [63]. The relationship between performance and boundary over-exploration at the end of the optimization process was assessed by means of an ordinary least squares regression between the degree of boundary over-exploration and logistically scaled performance using the OLS method of the *statsmodels* package (see figure 2(i) in the results for a visualization of the model) [69].

### 2.4. Assessment of boundary over-sampling

**Figure 3.**
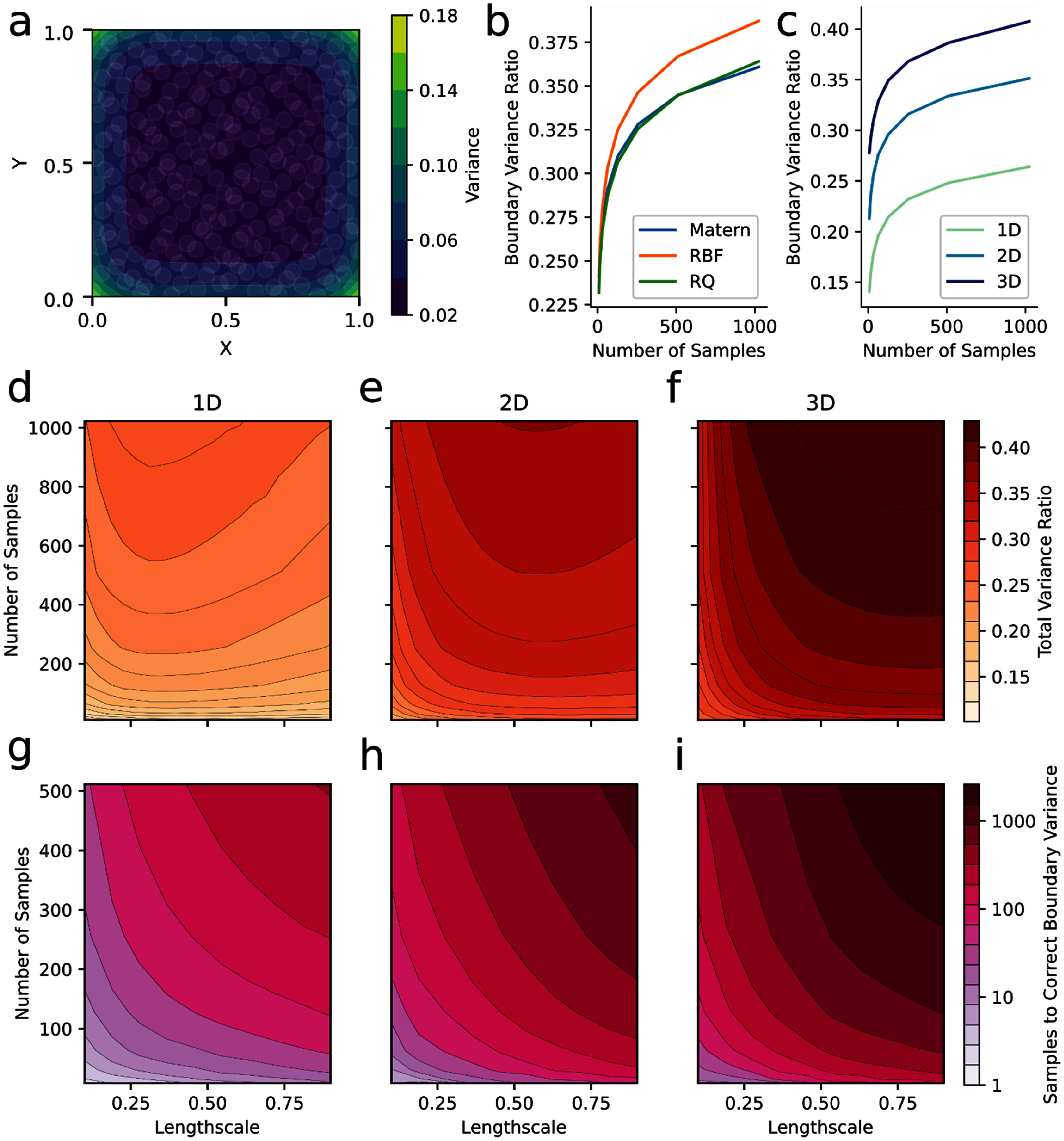
Effect of GP covariance kernel parameters on excessive boundary variance. (a) For roughly uniformly distributed samples (Sobol sequence), the posterior variance is disproportionately greater on the boundaries of the input space. (b) The extent of this disproportionality, measured as the ratio between the total variance on the boundary to the variance over the whole space (boundary variance ratio), grows as the number of samples increases for standard covariance kernels. (c) The boundary variance ratio is greater for increasing dimensions in the problem for all sample numbers. As a result, the total impact of this disproportionality is increased for higher dimensional problems. (d)–(f) Effect of sample count, kernel lengthscale, and problem dimensionality on the boundary variance ratio for a Matern kernel. Increasing sample count increased the ratio, increasing lengthscale increased the ratio up to a maximum, and the ratio was generally greater for higher dimensional problems. (g)–(i) The number of required boundary samples to reverse the disproportionate boundary effect (mean variance ratio < 1) as a function of sample count, kernel lengthscale, and problem dimensionality. Increasing samples, lengthscale, and dimensionality all led to more samples being required to reverse the effect.

The source of boundary over-sampling was assessed by a series of simulations using GPs with variable amounts of training data, kernel types, lengthscales, and dimensionality. Training data (number of samples) was sampled to fill the parameter space using a Sobol sequence in steps of powers of 2 ranging from 2 to 1024 using the *SobolEngine* class in *pytorch* (see figure 3(a) in the results for a visualization of typical training data) [70–72]. For kernel comparison, we considered the Matern 5/2, radial basis functions, and rational quadratic. Lengthscales for these kernels were generated by selecting 20 values linearly spaced between 0.1 and 0.9. We considered 1D, 2D, and 3D problems. The primary outcome for these simulations was the discrepancy between the variance on the boundaries and in the remainder of the parameter space. We determined this discrepancy by computing the ratio between the sum of the variance evaluated at points along the boundary of the space and the sum at regularly spaced points in the full parameter space. We term this the boundary variance ratio. We performed a second set of simulations where we computed how many samples were required to correct this discrepancy by sequentially adding samples at the highest variance locations on the boundary and recomputing the GP until the boundary variance ratio was less than one.

### 2.5. Boundary-avoiding Bayesian optimization

To prevent boundary over exploration during Bayesian optimization, we modified the GP by using an iterated Brownian bridge kernel (IBBK) and an input warp. IBBKs are two parameter kernels modeled after the Matern family with the key distinction that they are non-stationary [73]. While standard GP kernels have covariance functions that only depend on the distance between the points, the covariance of the IBBK is also a function of the location in parameter space. Specifically, the IBBK forces a covariance of 0 at the boundaries of the parameter space. Therefore, unlike standard kernels that have progressively more variance on the boundary of parameter space over the course of optimization, the lowest variance for the IBBK is always along the boundary. This has the added effect of making the GP’s posterior mean equal to a constant value along the boundary. Since most acquisition functions select samples based on a balance of mean and variance, these two effects will dissuade acquisition functions from sampling along the boundary. The two parameters of the IBBK, $\beta $ and $\varepsilon $ are balanced to localize the kernel and address the rate at which the kernel is warped towards the boundaries. For the purposes of this paper, we fixed these parameters to values of 20 and 50 respectively. These values were chosen because they represent a relatively tight length-scale which can describe a broad range of response surfaces. However, a smaller length-scale does run a greater risk of fitting to local noise. We show some comparative examples of GPs with various IBBK kernel parameters fit to example simulated data in supplemental figure 3.

**Figure 4.**
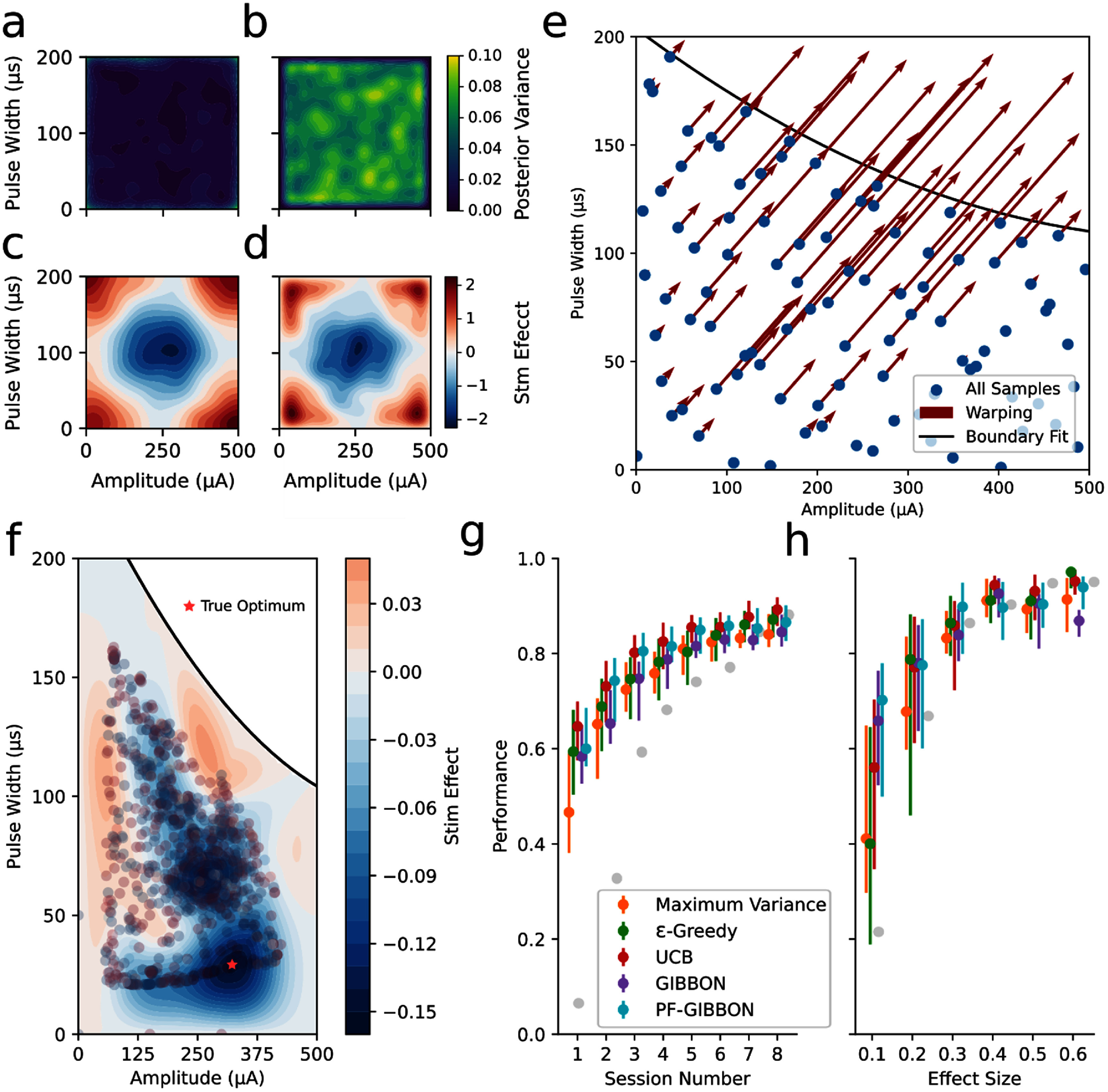
Bayesian optimization performance with boundary avoidance and input warping. (a)–(d) Illustration of the iterated Brownian bridge kernel (IBBK) for boundary avoidance. (a) A standard Matern kernel has the highest variance on the boundary of the parameter space. (b) In contrast, the IBBK has zero variance on the boundary of the parameter space. (c), (d) Both the Matern kernel (c) and IBBK (d) estimate a similar mean response surface but the IBBK forces the estimation to have zero mean at the boundary of the parameter space (visible as a small strip of white adjacent to the boundary). (e) Input warping is used to shift the safety boundary to the actual boundary of the parameter space. Points on the opposing boundary are not warped at all while those closest to the boundary are warped the most. (f) Example completed optimization for the same problem shown in figures 2(a) and (f) demonstrating that the IBBK and input warping prevent boundary sampling and result in more accurate identification of the optimal parameters. (g), (h) Performance of Bayesian optimization for five different acquisition functions after applying IBBK and warping corrections. Performance was calculated as the ratio between the effect of the learned optimal parameters to the ground truth optimal parameters. The maximum performance across all acquisition functions for standard approaches is indicated by the gray points. (g) Performance increased with the number of completed sessions with all acquisition functions reaching above 80% performance by end of the eighth session. (h) Performance was still reduced for low effect size problems especially for e-Greedy and variance maximization but the variants of the GIBBON acquisition function had performance above 70% even for the lowest effect size.

While the IBBK prevents excessive variance along the *actual* boundaries of the parameter space, it cannot do the same for a boundary *within* the parameter space, like a safety boundary. To counteract this type of over-exploration, we applied a warping function to the input data of the GP that shifted the safety boundary to the actual boundary of the parameter space (example shown in figure 4(e) and a labelled illustration of the method is included in in supplemental figure 4). Warping functions have previously been used in other Bayesian optimization applications to address non-stationarity [74]. To begin, we determined the intersection of the safety boundary with the upper (${x_1}$) and right boundaries (${y_1}$). Using these points, we found the location (${x_{\text{o}}})$ along the safety boundary where the path lengths on either side of the point were equal:

\begin{align*}&amp; \frac{{{{\mathop \smallint \nolimits}}_{{x_1}}^{{x_o}}\sqrt {1 + {{\left( {{\text{d}}y/{\text{d}}x} \right)}^2}} {\text{d}}x}}{{{{\mathop \smallint \nolimits}}_{{x_o}}^{500}\sqrt {1 + {{\left( {{\text{d}}y/{\text{d}}x} \right)}^2}} {\text{d}}x}} = \frac{{500 - {x_1}}}{{200 - {y_1}}}\end{align*}

\begin{align*}&amp;\qquad\quad {\text{d}}y/{\text{d}}x = \frac{{2\left( {x - h} \right)}}{a}\end{align*}

The warp direction ($m$) is then the slope of the line connecting ${x_{\text{o}}}$ to the upper right corner $\left( {500,\,200} \right)$ of the parameter space. A similar procedure could be used in higher dimensional spaces by instead using a surface area integral (3D) or volume integral (4D) for the corresponding higher dimensional safety bounds in those spaces.

Subsequently, sampled points could be warped into an adjusted parameter space. For each parameter pair $\left( {{x_i},{y_i}} \right)$, we calculated the intersection of the line containing the point and having slope $m$ with the safety boundary. Parameters that had a point of intersection outside the bounds of the parameter space were not warped. Parameters with intersection points to the left of ${x_{\text{o}}}$ were warped towards the upper boundary while those to the right were warped towards the right boundary. The warping distance $\left( {{d_{\text{w}}}} \right)$ was then determined with the following equation:

\begin{equation*}{d_{\text{w}}} = {D_{\text{s}}}\frac{{{D_{\text{p}}} - {D_{\text{o}}}}}{{{D_{\text{p}}}}}\end{equation*}

where ${D_{\text{s}}}$ is the length of the segment passing through $\left( {{x_i},{y_i}} \right)$ and meeting the opposing boundaries, ${D_{\text{o}}}$ is the distance from the lower boundaries to $\left( {{x_i},{y_i}} \right)$, and ${D_{\text{p}}}$ is the length of the segment passing through $\left( {{x_i},{y_i}} \right)$ between the lower boundaries and the safety boundary. The coordinates $\left( {{x_*},{y_*}} \right)$ of the parameters in the warped space can then be computed as follows:

\begin{equation*}{x_*} = {x_i} + \frac{{{d_{\text{w}}}}}{{\sqrt {{m^2} + 1} }}\end{equation*}

\begin{equation*}{y_*} = {y_i} + \frac{{{d_{\text{w}}}m}}{{\sqrt {{m^2} + 1} }}.\end{equation*}

This process forces points along the safety boundary to be warped to the upper boundaries of parameter space while points on the lower boundary are not warped at all. Those in between are warped more or less depending on how close they are to the safety boundary. Together with the IBBK, this leads to 0 variance along the safety boundary as well as the true boundaries of parameter space.

Bayesian optimization was performed similarly as in the standard simulations. However, there was no longer a nonlinear constraint since the warped parameter space covered the full range of input values. Additionally, since we kept the hyperparameters of the IBBK fixed, there were no other hyperparameters to optimize with these simulations

## 3. Results

### 3.1. Effect size meta-analysis

To assess the kinds of problems that could be addressed using Bayesian optimization in neurological and psychiatric applications, we conducted a literature search of typical within-subject effect sizes in the field. Within-subject effect sizes would indicate the size of the change from the intervention relative to the variability in the outcome, to which any optimizer would need to be robust. Our search revealed many studies that reported within-subject changes due to stimulation or drug-based interventions, however, very few reported the necessary information (trial-wise standard deviations) to actually compute a within-subject effect size. Our search identified 42 studies reporting sufficient information to calculate a within-subject effect size (figure 1(c)). Effect-sizes ranged from 0.087 to 2.016 with an average of 0.418 ± 0.342 (±standard deviation), which constitutes a small effect size according to standard descriptors [33]. The largest effect sizes were reported for physiological outcomes (0.689 ± 0.525) while the smallest were reported for reaction time (0.271 ± 0.145). Across the board, these studies indicate that any within-subject optimization would have to contend with a substantial amount of noise.

### 3.2. Simulation of standard Bayesian optimization

Using the range of effect sizes from our literature search, we conducted a set of simulations to assess the ability of standard Bayesian optimization to identify the optimal parameters in a standard problem. We modeled a two parameter (amplitude and pulse width) stimulation optimization problem for effect sizes ranging from 0.1 to 0.6 with the goal of identifying the minimum value in the response surface. We generated smooth, simulated surfaces with substantial variability in peak locations and shape including local maxima and minima (supplemental figure 2). Surfaces also included a safety boundary as any optimization of neuromodulation would have to avoid excessive dosage [62]. The response outcome was fixed to 0 at the left, bottom, and safety boundaries to model no dosage or aversive response (figure 2(a)). Bayesian optimization would sequentially sample the space based on a combination of the estimated mean response and variance (figures 2(b) and (c)). Over the course of 8 simulated sessions, all tested acquisition functions showed increasing performance with a maximum performance of 0.886 for GIBBON (figure 2(d)). All acquisition functions outperformed random selection by the eighth session. Maximum variance, a purely exploratory acquisition function, performed the worst, demonstrating that the exploitation–exploration trade-offs of the other acquisition functions were effective. All acquisition functions achieved performances above 80% for effect sizes greater than 0.3 (figure 2(e)). However, for an effect size of 0.1, only GIBBON and *ϵ*-greedy performed better than random selection after 8 sessions of optimization. Performance for an effect size of 0.2 was mediocre with the best acquisition function, GIBBON, only having a performance of 0.7. For reference, roughly a third of the studies in our literature search had effect sizes below 0.2 (figure 1(c)). After looking at the sampling behavior of our optimization, we noticed that there was substantial over-exploration of the boundaries of parameter space even for the acquisition functions that performed well (figure 2(f)). This over-exploration resulted in poor estimation of the response surface and relative under-sampling of potentially informative regions of parameter space. Additionally, in the context of an optimization problem for a neurological or psychiatric treatment, these samples would have been predominantly ineffective (zero dose) or actively harmful (provoking aversive effects when sampled). We investigated whether this over-sampling depended on the choice of acquisition function or optimization course and found that the worse performing acquisition functions (UCB and maximum variance) had a substantially greater proportion of samples on the boundaries (figure 2(g)). The degree of this over-sampling dropped over the course of the 8 sessions for all acquisition functions. Over-sampling also depended on effect size, with the largest degree of over-sampling occurring for the lower effect sizes for all acquisition functions except maximum variance (figure 2(h)). To determine if there was a direct relationship between over-sampling and optimization performance, we ran a regression between the two quantities for the final session of optimization across all acquisition functions. Over-sampling was significantly associated with poor optimization performance (*β* = −0.105, *p* < 7.08 × 10^−50^, *R*^2^ = 0.168) with performance rapidly falling off as the degree of over-sampling increased.

### 3.3. Assessing boundary over-sampling

To determine why over-sampling occurred with Bayesian optimization, we assessed the behavior of the underlying GP surrogate as the parameter space was filled with samples. We observed that for a uniformly filled parameter space, posterior variance was orders of magnitude greater on the boundaries of the space (figure 3(a)). This discrepancy in variance would result in most common acquisition functions almost exclusively selecting points on the boundaries when choosing exploratory samples. We investigated this variance effect by a series of simulations where we varied the GP kernel family, number of training samples, problem dimensi onality, and kernel lengthscale. The severity of the effect was evaluated by computing the ratio between the total variance on the boundary to the total variance in the remainder of the space (boundary variance ratio). For comparable lengthscale in a 2D problem, three common kernel families (Matern, RBF, and RQ) all showed increasing boundary variance ratio as the number of training samples increased (figure 3(b)). The boundary variance ratio also depended on dimensionality, with the 3D simulations having the greatest values across variable amounts of training data (figure 3(c)). These effects were also present across all lengthscales, with larger lengthscales generally showing greater boundary variance ratios regardless of the problem dimensionality or the amount of training data (figures 3(d)–(f)). Together, these results indicate that the excessive variance on the boundary is an innate feature of using GPs with smoother surfaces, and that it increases with problem dimensionality. Lastly, we assessed how many samples on the boundary would be required to balance the variance on the boundary and the remainder of parameter space. As the amount of training data grew, and for larger lengthscales, an exponentially larger number of boundary samples were required for balancing (figures 3(g)–(i)).

### 3.4. Simulation of boundary-avoiding Bayesian optimization

To account for the excessive variance on the boundary of parameter space, we adjusted the GP to use an IBBK and an input warp. Whereas a standard stationary kernel like the Matern family has the greatest variance on the boundary of parameter space (figure 4(a)), the IBBK has 0 variance on the boundary (figure 4(b)). This leads to the optimizer finding no exploratory value for samples on the boundary. Additionally, the Matern kernel and IBBK estimate the response surface comparably (figures 4(c) and (d)) but the IBBK forces a constant mean along the boundary of the parameter space visible as a white line along the border in figure 4(d). For a GP with 0 mean, this also leads to no exploitative value for points on the boundary. While the IBBK prevents the excessive variance on the boundaries of parameter space, it would not prevent similar variance inflation along a safety boundary. To address this, we warped the inputs to the GP to shift points along the safety boundary to the upper-right edges of the parameter space (figure 4(e)). Together, these measures dissuade the optimizer from selecting points near the boundaries leading to a more effective balance of exploration and exploitation in informative regions of parameter space and more accurate estimation of the response surface (figure 4(f)). The boundary avoiding optimization substantially improved performance across all 8 sessions for all acquisition functions (figure 4(g)). This improvement was particularly apparent early in the optimization process, where the best performance with boundary avoidance was 0.647 compared to 0.073 without. Boundary avoidance also substantially improved performance for problems with smaller effect sizes with PF-GIBBON having a performance of 0.702 using boundary avoidance compared to a maximum of 0.223 without for an effect size of 0.1.

## 4. Discussion

Identifying the optimal treatment parameters on an individual basis is the central focus of precision medicine and a promising avenue for improving therapeutic efficacy. However, especially in neuromodulation, substantial noise processes complicate efforts to identify robust relationships between treatment parameters and efficacy. We have demonstrated that standard approaches using Bayesian optimization for identifying this individualized parameter-response relationship fail for problems with relatively low signal to noise ratio. From our literature review, these noise levels are not uncommon and resolving this failure is necessary to ensure optimization methods are sufficiently robust for use in a clinical setting. Through a series of simulations, we identified that excessive variance on the boundaries of parameter space is the primary source of this poor performance. That excessive variance is an inherent feature of the standard kernels used in GP surrogates. We showed that this excessive variance can be addressed on the boundaries of parameter space and safety bounds via the combination of a non-stationary IBBK and input warp. These measures widen the space of problems that can be addressed by Bayesian optimization, improving the future potential for precision medicine in neurology and psychiatry.

In our boundary avoidance simulations, UCB was the overall best performer. However, PF-GIBBON was more effective at lower effect sizes. Srinivas *et al* extensively described regret bounds in their original paper proposing UCB which we leveraged in our implementation [19]. The same rigorous analysis has not been performed for GIBBON (partially for standard max-value entropy search) nor Pareto-front sampling [20–22]. The improvements of PF-GIBBON over standard *ϵ*-greedy and GIBBON suggest that there may be problems or points in the optimization progression where the two approaches complement each other. While we fixed the probability of using either method to a constant value, it is likely that this weighting could be optimally adjusted over the course of optimization as is done for the ${\beta _t}$ term in UCB. This kind of hyperparameter optimization is beyond the scope of this paper but would be important for maximizing performance in future applications.

We identified that the boundary over-sampling hindering the performance of standard Bayesian optimization was primarily caused by excessive variance on the boundary. This largely results from points in the center of parameter space having many nearby prior samples whereas those on the boundary lack neighbors beyond the boundary. As a result, the posterior variance drops more rapidly for samples within parameter space than those on the boundary. This effect is amplified by the increasing surface to volume ratio that happens in higher dimensions, which is a well-documented concern when applying Bayesian optimization in many dimensions [75]. Increasing lengthscales will also worsen this variance discrepancy since an even greater number of points will share variance within parameter space compared to those on the boundary. As many outcomes in neurology and psychiatry have smooth responses to parameter changes [16, 76], this lengthscale effect is particularly problematic without corrective measures.

Aside from the IBBK, other methods have previously been tested for reducing over-sampling of the boundaries in Bayesian optimization. One approach that is often suggested involves augmenting the training data with reflected copies of samples near the boundaries. This technique is commonly used in kernel density estimation for correcting the edges of bounded kernels [77]. Unfortunately, with GPs, this technique incurs a large computational cost by adding a substantial number of samples which is especially problematic as the problem dimensionality and lengthscale increase. Another similar approach involves augmenting the GP with virtual samples from the derivative when the acquisition function would suggest a sample on the boundary [78]. These virtual derivatives are directed to disincentivize sampling of the boundary. While powerful, this technique still increases the amount of training data in the problem. Further, this derivative based sampling approach is not well supported in the majority of existing packages for Bayesian optimization and GPs. The cylindrical kernel, another non-stationary kernel, has also been used to focus sampling during Bayesian optimization on the central portion of parameter space rather than the boundaries [75]. However, this technique still can have issues with excess variance on a hyper-spherical region within parameter space rather than on the boundary itself.

While the IBBK was effective in our simulations, it is not without issues. In cases where the boundaries of parameter space have been selected to include the full range of ineffective to aversive parameters, the IBBK’s 0 variance and a constant mean on the boundary is desirable as it will disincentivize boundarysampling. However, should an optimum exist on the boundary of parameter space, the IBBK would be unable to identify it. This could of course be addressed by widening the bounds but nevertheless presents a point of concern when applying the technique. Additionally, the hyperparameters of the IBBK are discrete making optimization more difficult compared to other kernel families [73]. It is possible that other more flexible kernels could be developed with properties similar to the IBBK, with kernels inspired by the beta distribution being a particularly promising option [79].

All the alternative boundary avoidance methods are unable to handle boundaries within parameter space. These boundaries are especially important for clinical applications because they can be used to model safety cut-offs, dosage limits, or hard ranges on combinations of device parameters [62]. By warping our safety boundary to the actual bounds of parameter space, we were able to take advantage of the IBBK’s boundary avoiding features to disincentivize sampling on this boundary as well. Our chosen strategy for input warping resembled a ‘shearing’ effect which seemed to cause minimal distortion in the response surface. This choice is still somewhat arbitrary and other warps that would concentrate the bound at a single point or edge could be more appropriate in other settings. It is likely that the warp could have the added benefit of improving the stationarity of the GP as the effects of typical interventions are often more sensitive to parameters near safety boundaries [62].

The performance of Bayesian optimization tends to suffer as the number of parameters grows. Especially in the neurostimulation space, this poses a problem as clinicians need to select a number of parameters (site, frequency, amplitude, pulse width, waveform, etc.) which quickly becomes a combinatorial explosion. For the purposes of our simulations, we focused on a 2D case with fixed output variance because we had substantial prior literature to inform the shape of our response surfaces. 3D problems addressing parameters beyond amplitude and pulse width have been underexplored for psychiatric and neurological applications, because the effects of frequency are generally poorly understood and may be non-linear. Similarly, combining the optimization here with optimization of stimulation site or waveform would require a mixed continuous-discrete optimizer. In less established test cases, output noise would likely have to also be estimated during optimization, requiring additional sessions or trials for a robust estimate of an effect. In practice, a hierarchical optimization process would likely be a promising solution where a range of reasonable parameters could be identified by ‘big data’ approaches which would subsequently be refined via Bayesian optimization. Strong prior probabilities from past patients would assist in regularization and enable personalization of parameters without the impossibility of searching for a needle in a therapeutic haystack.

## 5. Conclusion

In summary, we have demonstrated that with the proper considerations, Bayesian optimization can be effectively used for the high noise problems typically observed in neurological and psychiatric applications. This will hopefully assist in the development of optimization approaches for personalizing therapies in this field and improving patient outcomes. Future work will focus on refining these algorithms and determining the unique modifications that may be required in individual treatment contexts.

## Data Availability

The data and code that support the findings of this study are openly available at the following URL/DOI: https://github.com/tne-lab/NeuroOptimizationPaper.
